# Synthesis of clinical practice guidelines and recommendations for breast cancer survivorship care: a systematic review

**DOI:** 10.1007/s00520-026-11006-0

**Published:** 2026-07-23

**Authors:** Allison Brandt Anbari, Linda Anderson, Rebecca Graves, Elizabeth A. Anderson, Jennifer M. Hulett

**Affiliations:** 1 Sinclair School of Nursing, University of Missouri, 915 Hitt Street, Columbia, MO 65212 USA; 2 J. Otto Lottes Health Sciences Library, University of Missouri, Columbia, MO 65212 USA

**Keywords:** Breast cancer survivorship care, Primary care, Clinical practice guidelines

## Abstract

**Purpose:**

Breast cancer survivorship care is complex and not always provided according to standards or guidelines. Likewise, current cancer survivorship care interventions and programming do not always underscore clinical practice guidelines (CPGs) in the same fashion as interventions for other conditions. A call for improvement in breast cancer survivorship care warrants re-evaluation of current breast cancer survivorship care CPGs. The purpose of this systematic review was to (1) characterize CPGs that include content clinically relevant to people with a history of breast cancer and (2) appraise the possibility of consolidating current CPGs to simplified topics to be presented as a potentially clinically applicable model of breast cancer survivorship care (*PROSPERO CRD42024591381*).

**Methods:**

We conducted searches within six databases (PubMed, CINAHL, Scopus, PsycINFO, Cochrane DSR, and Cochrane Central), and in ProQuest Dissertations & Theses Global, ClinicalTrials.gov, and FigShare, Google, Google Scholar, and Bing. We located 3041 records to be screened and 191 records for full-text screening. Based on inclusion criteria considering clinical relevance to people with a history of breast cancer and AGREE II assessments, 21 records were included. We developed a codebook using the NCCN guidelines for cancer survivorship care.

**Results:**

Areas of concordance were identified as three consolidated topics: risk for recurrence, monitoring for new cancer, and fertility, reproductive, endocrine, and sexual health. These are underpinned by care coordination. Topics include main ideas derived from NCCN guidelines. We labeled each recommendation with main ideas that are more succinct, yet still inclusive/indicative of the full array of CPGs.

**Conclusion:**

We propose consideration and testing of our consolidated breast cancer survivorship care topics and main ideas. We hypothesize that our model could simplify breast cancer survivorship care provision in primary care settings.

## Introduction

There are approximately 4.3 million people with a history of breast cancer (PHBC) living in the USA [1], yet there is no widely adopted standard or model for breast cancer survivorship care delivery [2–8]. Cancer survivorship begins with diagnosis, continues through treatment, and extends indefinitely [8, 9]. To date, cancer survivorship assessments, planning, and interventions for care for PHBC remain piecemealed and tend to focus on singular domains such as surveillance for new cancers or recurrence or the long-term side effects from treatment [10]. Cancer survivorship care planning and education remain mostly focused on the risk for breast cancer recurrence and new primary cancers, inadequately addressing this group’s high level of supportive care needs [11], fear of recurrence [12, 13], and the long-term impact cancer survivorship can have on quality of life and patient outcomes [14].

Clinical practice guidelines (CPGs) for cancer survivorship care, including some that are breast cancer-specific, are in circulation [15–17]. However, current cancer survivorship care interventions and programming do not always present standards of care to patients and clinicians as clearly or consistently as guidelines for other chronic or complex conditions such as those for diabetes and hypertension [18]. Along the same lines, access to information about guideline-concordant cancer survivorship care can be limited. For example, recommendations listed on uptodate.com for management of hyperglycemia for adults with diabetes type II are open access while information about cancer survivorship care requires a subscription [19]. Equally problematic is that cancer survivorship care is not traditionally a component of medical or nursing training [20–23]. Our previous research has shown that the knowledge and application of CPGs in cancer survivorship care is limited and not automatic [20, 21].

The content within published cancer survivorship CPGs specifically for breast cancer and PHBC is complex [24]. Regardless of their origin or delivery format, we typically see CPGs and recommendations categorized into five breast cancer survivorship clinical topics or domains: (1) assessment and management of physical and psychosocial long-term and late effects; (2) health promotion; (3) surveillance for breast cancer recurrence; (4) screening for new primary cancers; and (5) coordinating care [15, 25]. The potential intersection or redundancy of these domains is not frequently mentioned; thus, the opportunity to synthesize or streamline is missed.

While this systematic review and synthesis are not about the use of cancer survivorship care plans, we would be remiss not to mention their role in CPG-concordant care. At one time, cancer survivorship care plans were unequivocally endorsed by leading organizations [15, 26, 27]. The care plan format, either paper or embedded in an electronic health record, was created to improve communication among cancer survivors and clinicians. Cancer survivorship care plans were also intended to facilitate transition from oncology to primary care by including clinical practice guidelines and recommendations pertaining to follow-up planning and cancer surveillance [2]. Addressing the recommendations can be cumbersome because of time and reimbursement limitations, the sheer number of recommendations, and also because of the potential complexities of monitoring very specific side effects from treatments [15, 28, 29]. Cancer survivorship care plans and their usefulness in facilitating cancer survivorship care are under evaluation because their outcomes are inconsistent and hard to measure [4, 28–34].

Similarly, the interface between oncology-led and primary care-led cancer survivorship care is complicated because their overlap and boundaries remain unclear. The number of PHBC continues to increase while the oncology workforce and capacity are beginning to decline. As such, it seems inevitable that primary care-led breast cancer survivorship care would become the standard. Recent work, including often cited systematic reviews [35, 36] and an overview of systematic reviews [37], points to two seemingly disparate themes. The first is a theme of barriers to primary care-led models within certain cancer survivorship domains, stemming from communication and coordination issues combined with a lack of readiness and resources. The other theme is that primary care-led cancer survivorship care has not been found to be inferior to oncology-led care, yet the former remains the exception. Thus, perhaps it could be argued that delivery of primary care-led breast cancer survivorship care is uncommon, not because primary care is unsuitable, but rather that the correctly designed approach for this space has not been fully realized.

In summary, a call for cancer survivorship care improvement warrants a re-evaluation of current planning techniques. Prevalence of chronic medical conditions such as hypertension, diabetes, and morbid obesity among people with a history of cancer has increased over the last 20 years [38]. Thus, it is likely that breast cancer treatment sequelae will be managed with comorbidities by different clinician specialists, not just oncology. We posit that long-term and late effects of breast cancer treatment can be monitored, assessed, and/or treated while also remaining vigilant in surveillance for new or recurring cancers. Viewing breast cancer survivorship care from a potentially adjusted stance could possibly streamline the use of CPGs in clinical practice and improve PHBC satisfaction with their care. To do so, there must be a thorough assessment and understanding of current CPGs clinically relevant to PHBC. To our knowledge, no systematic review or synthesis has recently evaluated CPGs relevant to PHBC. To address this gap and examine the possibility of streamlining breast cancer survivorship care, we conducted a systematic review to:

1. Characterize the nature of clinical practice guidelines (CPGs) that are clinically relevant to PHBC and breast cancer survivorship.
2. Examine the possibility of synthesis by cross-referencing and cross-matching the published CPGs to connect/consolidate them to simplified topics. If possible, execute the process and report the findings as a potentially clinically applicable model for breast cancer survivorship care in primary care settings.

## Methods

### Approach

Systematic reviews of CPGs are often conducted to understand the nature of CPGs and potentially “identify gaps in knowledge about the current state of clinical guidance on a particular issue” [39]. Systematic reviews of CPGs should be conducted with the same rigour and core components as systematic reviews of general topics. For our review, we used guidance provided by Johnston et al. [39] combined with general systematic review procedures as described by the Joanna Briggs Institute [40]. Likewise, we report here our process and findings using PRISMA reporting recommendations [41]. At the onset, we registered our protocol with PROSPERO (*CRD42024591381*) and followed the plan as written, with one amendment. Institutional Review Board approval was not needed for this systematic review of published or publicly available content.

### Data collection

Guided by Johnston et al. (2018), we created a PICAR (**p**opulation, **i**ntervention, **c**ontent, **a**ttributes, **r**ecommendation characteristics) table (see Table 1). The PICAR approach is informed by the traditional PICO(T/S) (**p**opulation/problem, **i**ntervention, **c**omparison, **o**utcome, and **t**imeframe, setting) framing used for systematic reviews but is specific to systematic reviews of CPGs. The R in PICAR refers to CPG and recommendation inclusion criteria.

**Table 1:** Literature retrieval plan based on problem, interventions, content, attributes, and recommendation characteristics (PICAR) [39]

PICAR	
P = population, clinical indications, conditions	Adult (>18 years) outpatients; cancer survivors (accept definition of CPG issuer); breast when possible
I = interventions	Survivorship care guidelines; breast specific when possible; general clinically relevant guidelines to PHBC if issuer does not have breast specific
C = content	Cancer survivorship after acute treatment is completed, also referred to as follow-up care
A = attributes of eligible CPGs	Language: available in English Year: 2006 (rationale is publication date of “From Cancer Patient to Cancer Survivor: Lost in Transition” [26] Publishing region: North America, Europe, Japan, Australia, New Zealand Version: latest version only; breast version when available; survivorship/PHBC relevant content, no duplicates Development process: must be explicitly evidence-based (flag any that might meet inclusion criteria otherwise for documentation purposes only) System of rating evidence: must use a system to rate the level of evidence behind recommendations Scope: primary focus on cancer survivorship care; breast when available, clinically relevant post-cancer treatment Recommendations: at least one eligible recommendation is reported and is clinically relevant to PHBC
R = recommendation characteristics	Levels of confidence: must be explicit (e.g., GRADE) Locating recommendations: locate CPGs, next isolate/itemize recommendations that correspond to prevention/identification of recurrence or new primary cancers

Our formal search strategy was twofold. First, we used the advanced search features of traditional search engines (i.e., Google and Bing) to locate breast cancer survivorship CPGs and recommendations that may or may not be listed within scholarly databases. Search terms included: (“breast cancer” OR “breast neoplasm” OR “breast carcinoma” OR “ductal carcinoma in situ” OR “lobular carcinoma in situ”) AND (“survivor” OR “survivors” OR “survivorship” OR “follow-up”). This initial search was similar to an environmental scan in which we identified CPGs already known to the team, such as those published by the National Comprehensive Cancer Network and the American Cancer Society.

**Fig. 1:**
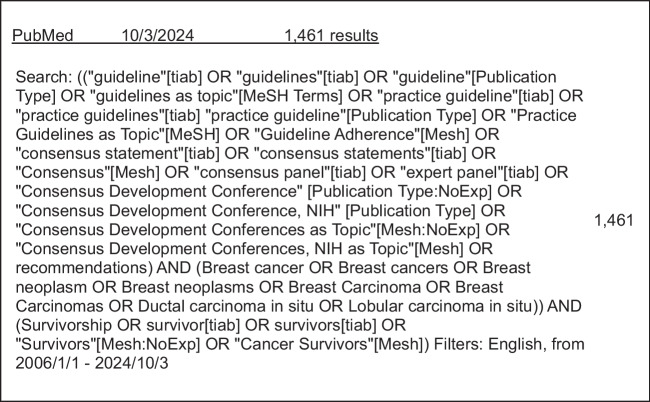
PubMed search strategy

Next, the health sciences librarian on our team (RG) created a search strategy of key terms for scholarly databases: PubMed, CINAHL, Scopus, Cochrane DSR, and Cochrane Central. We also searched Google Scholar, ProQuest Dissertations & Theses Global, ClinicalTrials.gov, and FigShare. An example of the PubMed search strategy is included in Fig. 1. We were able to confirm the appropriateness of our twofold search strategy when records known to the research team located in the environmental scan and identified as requisite were located. Through this formal search, we identified and collected the supplemental materials and other publications corresponding to the CPGs.

### Data analysis—inclusion/exclusion and data extraction

**Fig. 2:**
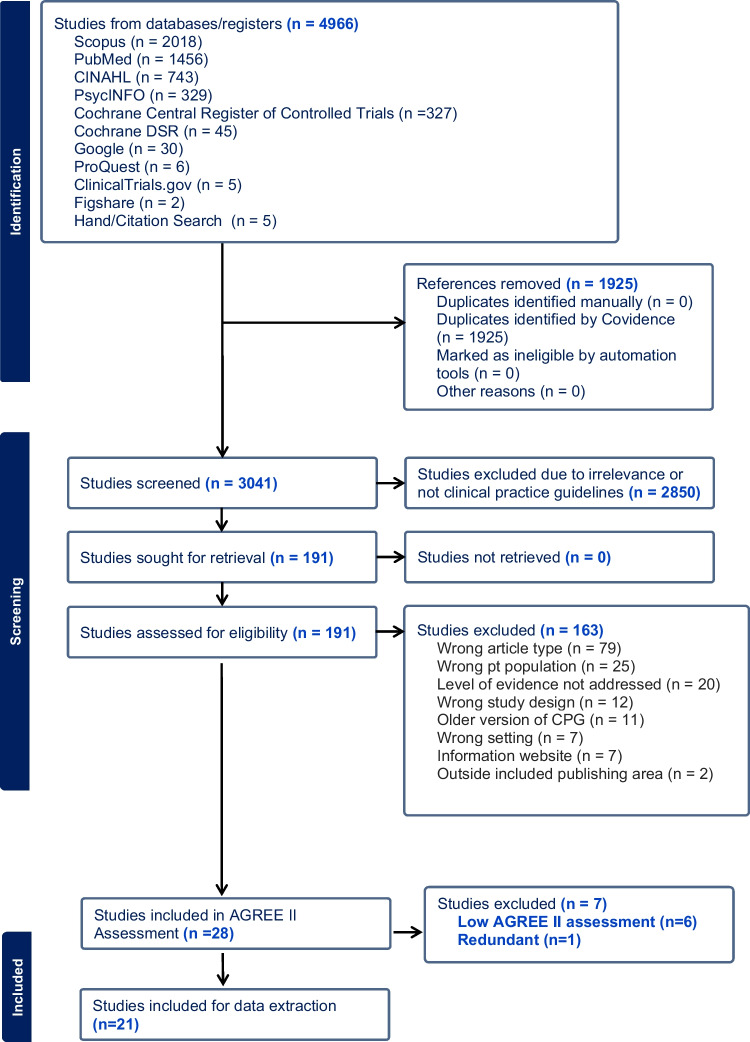
PRISMA flow diagram

Inclusion criteria were similar to the items listed as attributes in Table 1. Clinical practice guidelines were included if they were available in English and originated from North America, Europe, Japan, Australia, or New Zealand to accurately reflect clinical context [39]. We included only the most current versions of CPGs and published after the year 2006 or the same year “From Cancer Patient to Cancer Survivor: Lost in Transition” was published [26]. This publication was one of the first to outline key survivorship care domains and as such, is considered to be seminal work in the science of cancer survivorship [42, 43]. The way in which the CPGs were developed and approved must have been explicitly described and evidence-based within the CPG itself or in the corresponding supplemental documents. Likewise, a system of rating the level of evidence supporting each guideline and recommendation must have been included. While the focus for this paper is breast cancer, we included general cancer survivorship CPGs when breast-specific guidelines were not available or when the topic of recommendations was clinically relevant to PHBC (e.g., general cancer treatment sequelae such as fatigue, fertility). Guideline documents that included actual treatment for breast cancer were only included if they specified recommendations for cancer survivorship care. We also included guidelines if the documents used the terms “follow-up care” or “post-treatment care.” Otherwise, they were excluded. Twenty-eight records of CPGs met inclusion criteria at this step (see Fig. 2) [41].

### AGREE II assessments

The methodological quality of the included CPGs was evaluated using the validated Appraisal of Guidelines for Research and Evaluation II (AGREE II) rating system prior to full data extraction [44]. The AGREE II system was developed to evaluate the methodology contributing to guideline development and whether the methods were rigorous or reported. The assessment included 23 items within 6 different domains (scope and purpose; stakeholder involvement; rigour of development; clarity of presentation; applicability; and editorial independence), each rated on a scale from 1 to 7 (1 being the lowest and 7 being highest quality). The six individual domain scores were then converted to scaled domain scores, using the formula:

((Obtained score − Minimum possible score)/(Maximum possible score − Minimum possible score)) × 100

In addition to individual domain scores, there are two global rating items, the overall assessment score and the appraiser’s recommendation for use of the guideline. The overall assessment score is also rated on the 1 to 7 scale and is not derived from aggregating individual domain scores but is at the discretion of the appraiser. The appraiser’s recommendation for using the guideline is categorized as “Yes,” “Yes, with modifications,” or “No.”

Two reviewers (authors ABA and LA) assessed each CPG using a spreadsheet designed to track and total each domain score and an overall total (see Table 2 for AGREE II scores of included CPGs). During AGREE II assessment, any additional relevant supplemental files were located and saved for future review. If updated versions of the guidelines were located during the AGREE II assessment, we replaced the older version with the new (e.g., NCCN Adolescent and Young Adult (AYA) Oncology published in late 2024 and NCCN Cancer Fatigue Guidelines published in January 2025). Likewise, upon this initial review of the publications, we became aware of clinical practice guidelines that were redundant (*n* = 1), did not include information clinically relevant to PHBC (*n* = 4), or were the incorrect study format (e.g., a review of clinical practice guidelines (*n* = 4). These were added to the studies excluded at screening and the PRISMA flow chart was updated accordingly.

**Table 2:** AGREE II scaled domain scores and overall assessment of included guidelines

Author, year	Domain 1	Domain 2	Domain 3	Domain 4	Domain 5	Domain 6	Overall assessment	Recommend	
	Scope and purpose (%)	Stakeholder involvement (%)	Rigour of development (%)	Clarity of presentation (%)	Applicability (%)	Editorial independence (%)	(Max 7)	R1	R2
ACOG, 2021	100%	66.7%	100%	100%	100%	83.3%	6.7	Y	Y
Bhatia et al., 2024	100%	38.9%	54.2%	100%	95.8%	79.2%	5.4	YM	YM
Bower et al., 2024	100%	100%	100%	100%	100%	100%	7.0	Y	Y
Davies et al., 2020	100%	55.6%	87.5%	100%	18.8%	83.3%	5.5	YM	YM
Freedman et al., 2021	100%	100%	93.8%	100%	58.3%	100%	6.4	Y	Y
Gradishar et al., 2025	100%	94.4%	100%	100%	100%	100%	7.0	Y	Y
Hassett et al., 2020	100%	100%	100%	100%	100%	87.5%	6.9	Y	Y
Jacobson et al., 2021	100%	69.4%	96.9%	100%	87.5%	50.0%	6.3	Y	Y
Jankowski et al., 2025	100%	91.7%	100%	100%	97.9%	70.8%	6.8	Y	Y
Lambertini et al., 2020	100%	69.4%	100%	100%	100%	70.8%	6.6	Y	Y
Loibl et al., 2024	100%	72.2%	100%	97.2%	95.8%	83.3%	6.6	Y	Y
Lyon et al., 2022	100%	94.4%	87.5%	100%	100%	75.0%	6.6	Y	Y
Paluch-Shimon et al., 2022	100%	100%	92.7%	100%	100%	83.3%	6.8	Y	Y
Riba et al., 2024	100%	100%	86.5%	100%	100%	83.3%	6.6	YM	Y
Rock et al., 2022	100%	72.2%	79.2%	91.7%	91.7%	50.0%	5.9	YM	Y
Runowicz et al., 2016	100%	100%	100%	100%	100%	87.5%	6.9	Y	Y
Shapiro et al., 2019	100%	100%	100%	100%	100%	100%	7.0	Y	Y
Su et al., 2025	100%	100%	100%	100%	100%	100%	7.0	Y	Y
Tozawa et al., 2022	100%	83.3%	87.5%	97.2%	62.5%	100%	6.2	Y	Y
Tsuji et al., 2025	100%	100%	87.5%	100%	97.9%	79.2%	6.6	Y	Y
Wong et al., 2024	100%	100%	100%	100%	100%	100%	7.0	Y	Y

Notes. *ACOG*, American College of Obstetricians and Gynecologists; *R1*, reviewer 1; *R2*, reviewer 2; *Y*, yes; *YM*, yes with modifications. Scaled domain percentages are calculated as ((Obtained score − Minimum possible score)/(Maximum possible score − Minimum possible score)) × 100. Overall assessment scores are rated on a scale from 1 (lowest possible quality) to 7 (highest possible quality)

The AGREE II guidelines do not specify a minimally acceptable domain score. As such, there is flexibility in how quality thresholds pertinent to the review are defined [44]. Given our goals of potentially synthesizing the CPGs and for the synthesis to be comprised high-quality CPGs, we elected to exclude CPGs with AGREE II Domain 3: Rigour of Development domain scores less than four out of seven. The Rigour of Development domain includes criteria such as were the CPGs systematically developed using sound search strategies, were the inclusion and exclusion criteria clear, were the recommendations externally reviewed, and whether “the health benefits, side effects, and risk have been considered in formulating the recommendations [44].” By evaluating domain criteria such as this, we excluded an additional six CPGs before the synthesis process. A total of 21 CPGs were moved forward for data extraction (see Table 2 for an AGREE II scaled domain scores and overall guideline assessment ratings of the included CPGs).

### Data extraction

**Fig. 3:**
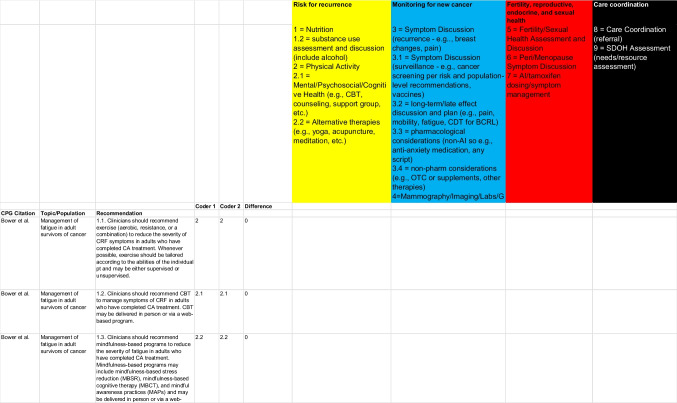
Sample of recommendation extraction spreadsheet—with topics and main ideas in first rows

Included CPGs were first saved as portable document files (pdfs) in Covidence (https://www.covidence.org) and then in a shared OneDrive folder for ease of access by the team. We familiarized ourselves with the included CPGs by exporting a bibliography from Covidence that was edited to only list the included records. An Excel© workbook facilitated standardized extraction from each set of CPGs (Table 3). Recommendations within each set of CPGs that were specific to breast cancer survivorship (i.e., clinically relevant to PHBC) were summarized and placed into the corresponding row (Fig. 3).

**Table 3:** Characteristics of included clinical practice guidelines

Author, year	Country	Title	Affiliate organization	Update of earlier guideline?	Evidence appraisal	Process
American College of Obstetricians and Gynecologists, 2021	USA	Treatment of urogenital symptoms in individuals with a history of estrogen-dependent breast cancer	ACOG	Yes	Expert consensus	Literature review/expert panel
Bhatia et al., 2024	USA	Adolescent and young adult (AYA) oncology (version 2.2025)	NCCN	Yes	NCCN Categories of Evidence and Consensus	Literature review
Bower et al., 2024	USA	Management of fatigue in adult survivors of cancer: ASCO–Society for Integrative Oncology guideline update	ASCO and SIO	Yes	Adapted from: Cochrane Risk of Bias tool, Physiotherapy Evidence Database Scale, GRADE	Literature review/expert panel
Davies et al., 2020	USA	Interventions for breast cancer-related lymphedema: clinical practice guideline from the Academy of Oncologic Physical Therapy of APTA	APTA	No	APTA’s Critical Appraisal Tool for Experimental Intervention Studies	Literature review
Freedman et al., 2021	USA	Individualizing surveillance mammography for older patients after treatment for early-stage breast cancer	ASCO and SIO	No	Expert consensus	Literature review/expert panel
Gradishar et al., 2025	USA	Breast cancer (version 4.2025)	NCCN	Yes	NCCN Categories of Evidence and Consensus	Literature review
Hassett et al., 2020	USA	Management of male breast cancer: ASCO guideline	ASCO	No	Expert consensus	Literature review/expert panel
Jacobson et al., 2021	Canada	Guideline no. 422f: menopause and breast cancer	SOGC	Yes	GRADE	Literature review
Jankowski et al., 2025	USA	Cancer-related fatigue (version 2.2025)	NCCN	Yes	NCCN Categories of Evidence and Consensus	Literature review
Lambertini et al., 2020	Italy	Fertility preservation and post-treatment pregnancies in post-pubertal cancer patients: ESMO clinical practice guidelines	ESMO	Yes	Adapted from the Infectious Diseases Society of America-United States Public Health Service Grading System	Literature review
Loibl et al., 2024	Germany	Early breast cancer: ESMO clinical practice guidelines for diagnosis, treatment and follow-up	ESMO	Yes	Adapted from the Infectious Diseases Society of America-United States Public Health Service Grading System	Literature review
Lyon et al., 2022	UK	2022 ESC guidelines on cardio-oncology developed in collaboration with the European Hematology Association (EHA), the European Society for Therapeutic Radiology and Oncology (ESTRO) and the International Cardio-Oncology Society (IC-OS)	ESC with EHA, ESTRO, and IS-OC	No	ESC Guidelines for Classes of recommendations and Levels of Evidence	Literature review/expert panel
Paluch-Shimon et al., 2022	International	ESO–ESMO fifth international consensus guidelines for breast cancer in young women (BCY5)	ESO, ESMO	Yes	ESC Guidelines for Classes of recommendations and Levels of Evidence	Literature review/expert panel
Riba et al., 2024	USA	Distress management (version 1.2025)	NCCN	Yes	NCCN Categories of Evidence and Consensus	Literature review
Rock et al., 2022	USA	American Cancer Society nutrition and physical activity guideline for cancer survivors	ACS	No	Expert consensus	Literature review
Runowicz et al., 2016	USA	American Cancer Society/American Society of Clinical Oncology breast cancer survivorship care guidelines	ACS/ASCO	No	ACS/ASCO collaboration; expert consensus	Literature review/expert panel
Shapiro et al., 2019	USA	Management of osteoporosis in survivors of adult cancers with nonmetastatic disease: ASCO clinical practice guideline	ASCO	No	Expert consensus	Literature review/expert panel
Su et al., 2025	USA	Fertility preservation in people with cancer: ASCO guideline update	ASCO	Yes	AMSTAR; Cochrane Risk of Bias tool; ROBINS-I; GRADE	Literature review/expert panel
Tozawa et al., 2022	Japan	Japan Society of Clinical Oncology clinical practice guidelines 2017 for fertility preservation in childhood, adolescent, and young adult cancer patients: part 2	JSCO	No	Expert consensus	Literature review/expert panel
Tsuji et al., 2025	Japan	Japan’s cancer survivorship guidelines for exercise and physical activity	GDOC	No	Cochrane Risk of Bias Tool; MINDS Manual for Developing Clinical Practice Guidelines 2017; expert consensus	Literature review/expert panel
Wong et al., 2024	China	Multinational Association of Supportive Care in Cancer (MASCC) clinical practice guidance for the prevention of breast cancer-related arm lymphoedema (BCRAL): international Delphi consensus-based recommendations	MASCC	No	Expert consensus	Expert panel

Notes. *ACOG*, American College of Obstetricians and Gynecologists; *ACS*, American Cancer Society; *AMSTAR*, Assessment Of Multiple Systematic Reviews; *APTA*, American Physical Therapy Association; *ASCO*, American Society of Clinical Oncology; *ESC*, European Society of Cardiology; *ESMO*, European Society for Medical Oncology; *ESO*, European School of Oncology; *GDOC*, Guidelines’ Development Oversight Committee; *GRADE*, Grading of Recommendations Assessment, Development and Evaluation; *JSCO*, Japan Society of Clinical Oncology; *MINDS*, Medical Information Network Distribution Service; *NCCN*, National Comprehensive Cancer Network; *ROBINS-I*, Risk of Bias in Non-randomized Studies–of Interventions; *SIO*; Society for Integrative Oncology; *SOGC*, Society of Obstetrician and Gynaecologists of Canada

### Synthesis process

Knowing that CPGs might often reference each other (we refer to this as cross-referencing) and/or be duplicative yet concordant (we refer to this as cross-matching), we pursued our second study objective by appraising the included CPGs’ and recommendations’ connections. Having fully evaluated the included CPGs and their topics (inductively) and using the guidelines and standards of general cancer survivorship care delineated in the NCCN Guidelines—Survivorship 2025 [16] as parameters for a model (deductively) (Fig. 3), overarching topics were discussed and drafted: risk for recurrence, monitoring for new cancer, fertility, reproductive, endocrine, and sexual health, and care coordination (Fig. 3). We then identified corresponding main ideas for each topic, staying within the parameters and recommendations of the NCCN standards, yet as specific to breast cancer survivorship care as possible. Main ideas were also derived from the NCCN standards [16] and were selected to serve as requisite assessments or discussion points to be considered when planning care for PHBC. For example, within the fertility, reproductive, endocrine, and sexual health topics are the main ideas of perimenopause and menopause discussions and aromatase inhibitors/tamoxifen dosing/symptom management.

We were motivated to use the NCCN set of standards and guidelines [16] as the polestar for topic and main idea generation as this set is typically considered to be the most general yet comprehensive and is more frequently and recently updated than the often cited and included American Cancer Society guidelines specific to breast cancer survivorship care published in 2016 [15]. These two documents also have overlapping content areas that are accordingly reflected in the overarching topics and main ideas we identified. Each topic and main idea can be connected back to one or both of these CPGs and remain driven by breast cancer-specific recommendations.

For synthesis and labeling purposes, main ideas were given a number within each topic (Fig. 3). Identical copies of the initial extraction spreadsheet were created to facilitate the synthesis process. Clarifications about the labeling process were discussed during team meetings prior to this step. Three research team members (ABA, EA, and/or JH) then independently went row by row, recommendation by recommendation, and assigned a number to the recommendation. Every recommendation (*n* = 406) was independently labeled with a main idea by two research team members. The other team members’ ratings were not visible to each other. Because the purpose of this process was to gauge the soundness of the topic areas and main ideas, we did not meet or discuss to try to reach consensus. Once ratings were completed, we immediately evaluated interrater reliability.

## Results

### Characteristics of included CPGs

Table 3 includes key attributes of the included CPGs. In summary and per inclusion criteria, all CPGs were reported after 2006, with the earliest set of CPGs published in 2016 [15]. The guidelines represented 10 affiliate organizations, with five guidelines representing more than one affiliate organization [15, 45–48]. Fifteen of the 21 CPGs were generated by organizations within the USA and Canada, five were Europe-based or international organizations [47, 49–51], and two were Japan-based organizations [52, 53]. Nine of the CPGs used expert consensus alone (*n* = 7) or in combination with another methods (*n* = 2) to assess and appraise the levels of evidence for their recommendations. Others used their organizations’ guidance (*n* = 7) or Cochrane (in combination) (*n* = 3) or the Infectious Diseases Society of America-US Public Health Service Grading System (*n* = 2).

Five of the 21 CPGs addressed general cancer survivorship care and included recommendations that were either specific to PHBC or all people with a history of cancer [15, 47, 50, 54, 55]. Sixteen of the 21 CPGs addressed specific conditions or concerns that are clinically relevant to PHBC: cardiovascular disease [46], distress [56], fatigue [45, 57], osteoporosis [58], physical activity [52, 59, 60], and menopause management [61, 62]. The target population for most CPGs was adults; however, three guidelines pertained to all age groups [57, 59, 63], three were limited to young adults [47, 53, 64], and one was limited to adults over age 75 [48]. Seven CPGs had recommendations for males as well as females [47, 49, 50, 53, 59, 63, 64] while one CPG exclusively pertained to males [55]. Three CPGs had recommendations that addressed gender diverse populations [47, 55, 64]. Across the included CPGs, we did not observe contradicting recommendations. This is likely because of the specificity of the CPGs to a specific clinical area (e.g., fatigue or lymphedema) or population (e.g., males or adults over age 75).

### AGREE II evaluation of the included CPGs

AGREE II does not calculate an overall quality score based on domain scores but relies on appraiser assessment of the quality of the CPG and its recommended use [44]. For this review, any differences in AGREE II key item scores of >2 points between the two appraisers (ABA, LA) were resolved through discussion and did not require third-party adjudication.

Overall, the quality of the CPGs included for synthesis was high, as those with low scores on Domain 3: Rigour of Development were excluded from the review and synthesis process, after careful analysis by the authors, who ensured no pertinent guidelines were missed. Because the methodologically questionable CPGs were removed early in the synthesis process, the subsequent consolidated model is not believed to have been influenced by the quality appraisal of individual included CPGs.

The highest rated AGREE II scaled domain scores were Domain 1: Scope and Purpose (mean 100%) and Domain 4: Clarity of Presentation (99.3 ± 1.9). The lowest rated domains were also those with the highest variability including Domain 2: Stakeholder Involvement (86.1 ± 18.1; range = 38.9 to 100) and Domain 6: Editorial Independence (84.1 ± 15.2; range 18.8 to 100). Regarding the Stakeholder Involvement Domain, the most common deficiency was the lack of explicit inclusion of the views and preferences of the target population. Regarding the Editorial Independence Domain, the most common deficiency was failure to report that the views of the funding body did not influence the CPG content. Four CPGs met 100% of the AGREE II criteria and were also given an overall assessment score of 7/7 [45, 51, 58, 63]. Only two CPGs met less than 80% of the AGREE II criteria: Bhatia et al. (2024) (78.0 ± 26.0) and Davies et al. (2020) (74.2 ± 31.6) and were assessed to “require modifications before recommending their use” by both appraisers. Their inclusion is not thought to risk impacting the quality of the overall synthesis because both met the initial and underlying Rigour of Development (Domain 3) inclusion criteria of having a score over 4 out of 7.

### Synthesis results

**Fig. 4:**
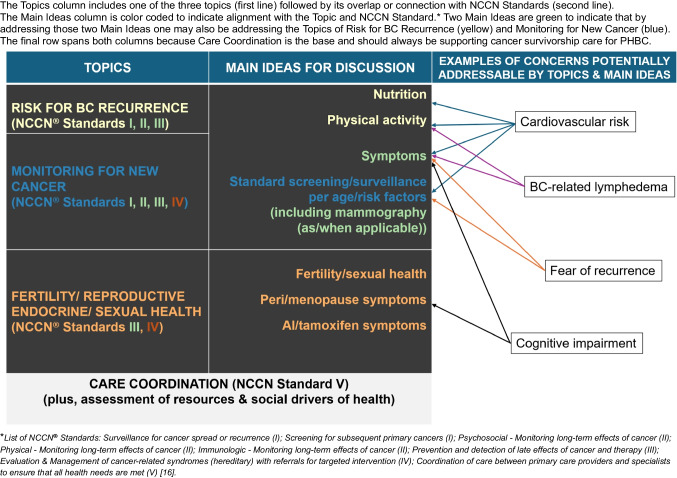
Diagram of potential application of topics (with corresponding NCCN standards) and main ideas

Recommendation labeling within our topics and main ideas model (Fig. 4) had an unweighted Cohen’s kappa (*k*) of 0.625 (95% CI 0.57 to 0.67) and a weighted Cohen’s kappa (*k*) of 0.696 (95% CI 0.65 and 0.75) indicating moderate to substantial agreement between raters [65]. Given that the weighted *k* was larger than the unweighted *k*, there was signal that labeling discordance was concentrated on closely related main ideas, as opposed to discordance across less closely related main ideas. For example, the most common disagreement between coders was within the same topic area of “monitoring for new cancer” between the main ideas of “symptom discussion” and “long-term/late effect discussion” (*n* = 18). These were later consolidated into the main idea of “symptoms” within our model.

The label “long-term/late effect discussion” was the most frequent cause of discordance (*n* = 80); however, those disagreements were most often within the same topic areas of “monitoring for new cancer” or the “risk for recurrence” signaling that some individual recommendations and main ideas reasonably might fit in multiple topic areas which supports the areas of overlap we later indicate in our model.

Given the Cohen’s kappa calculations and that many of the discordant main idea labels were within the same topic area, we met to further refine and polish the topics and main ideas so that they were inclusive but also presentable in a figure or model. Throughout post hoc discussions about the labeling process and how to present our findings, we noted the overlap of care coordination and how at times it could be unclear how to distinguish between it and another topic or main idea. Twenty-four instances of across-topic discordance included the main ideas of “care coordination” (*n* = 16) or “needs/resource assessment” (*n* = 8). We realized the potential to condense these topics and concluded that the fourth topic of care coordination essentially underpinned the other three topics and should be considered as foundational when addressing each of the three other topics. This process gave rise to our proposed model as shown in Fig. 4.

Figure 4 demonstrates the connection of the topics listed in the first column to NCCN standards (included as Roman numerals in the first column) and then how the main ideas (included in the second column) could potentially address examples of concerns a PHBC may discuss during a primary care visit (included in the third column). Main ideas are more detailed assessment areas to consider when addressing the topic areas; however, there is overlap in the main ideas that potentially contributes to the simplified approach and its usability in primary care. In Fig. 4, the topics are color-coded to match the main ideas. Of note, the two main ideas of “symptom discussion” and “mammography” are green to indicate their overlap with the two topic areas of risk for breast cancer recurrence (yellow) and monitoring for new cancer (blue) (yellow + blue = green). For example, during symptom discussions based on patient-reported concerns about recurrence or new cancers (a topic in the first column), a PHBC and their primary care clinician may identify risks for or symptoms of breast cancer-related lymphedema (a concern potentially addressable and presented as an example in the third column). These concerns may be addressed through additional discussion about symptoms and health promotion activities such as physical activity (a main idea in the second column). If symptoms such as swelling and mobility issues are present, a referral to lymphedema therapy (care coordination, the bottom row of Fig. 4, spanning entire figure as a foundation) would be needed. We believe this acknowledgment and incorporation of the overlap of the traditional cancer survivorship domains makes the model distinctive and perhaps more usable in primary care. The simplified structure potentially lends itself well to busy primary care settings and enables a natural flow of assessment and discussion when providing breast cancer survivorship care.

Care coordination and referrals for breast cancer survivorship care are analogous in our model but are at the base for a reason. Care coordination serves as a foundational fourth topic that underpins the other three topics which have corresponding main ideas to be considered. Many concerns and social needs could be potentially managed within primary care via care coordination until symptoms and screening warrant either additional workup, specialty care, or access to resources. This would not be dissimilar from primary care managing glucose control for a patient with type II diabetes until management became ineffective or additional side effects of diabetes called for an endocrinology consultation.

## Discussion

What if breast cancer survivorship care could be addressed by considering three topic areas supported by care coordination? Our systematic review, synthesis, and proposed model support a growing body of research that is attempting to consolidate, simplify, and/or polish cancer survivorship care recommendations so they are more accessible and amenable to primary care settings [66–69]. Through this systematic review of CPGs clinically relevant to PHBC, we came to believe that CPGs address areas that are broad but also very detailed, so much so that their implementation by clinicians is challenging. There is a signal that three topics—risk for recurrence, monitoring for new cancer, and fertility/reproductive endocrine sexual health—could potentially capture and streamline many of the recommendations clinically relevant to PHBC and be more user-friendly and less daunting. Underpinning and supporting the three topics is care coordination, where referral to other specialties and/or other resources is foundational in addressing access or resource concerns. Our synthesis results and corresponding model are different from existing breast cancer survivorship care models because the topics and main ideas are presented with primary care in mind. We propose a simplified approach to addressing cancer survivorship needs without overwhelming the nature of the visit. In synthesizing oncology-centric CPGs, we developed a primary care-centric translation intended to inform subsequent testing and intervention development.

An example of how the model’s three topics supported by care coordination could be potentially operationalized in primary care practice is presented here. Model components, that is, the topics and main ideas, are noted in parentheses. A 46-year-old PHBC, Katy, completed treatment for stage IIb triple negative breast cancer 5 years ago and is transitioning from oncology to primary care. At her annual wellness visit, Katy’s primary care clinician, Dr. V., incorporates the three topics as anticipatory guidance. They begin with recurrence risk (topic), including Katy’s understanding of her risk and strategies to reduce it. Katy perceives her risk to be low given the 5-year window. Discussion also includes potential late effects (main idea, symptoms), such as her reported left arm/shoulder mobility limitations. She declines a referral to physical therapy at this time (care coordination) with encouragement to follow-up if symptoms worsen. This prompts discussion of health promotion, including gradually increasing physical activity (main idea, e.g., yoga, walking) and improving nutrition (main idea) by reducing fat intake and alcohol consumption. These behaviors relate to breast cancer recurrence risk and monitoring for new cancers (topics) and provide an entry point to discussion of fertility and sexual health (topic). When Dr. V. invites Katy to address concerns, Katy reports some body image concerns, but declines the need for additional resources. Dr. V. plans to revisit this at a future appointment. Dr. V. reinforces that these health promotion behaviors (main ideas) also reduce risk of new primary cancers (topic) and transitions to review the recommended cancer screenings (main idea). Katy is up to date on mammography and scheduled for MRI next month. Dr. V. plans to confirm results. They also review the need for colonoscopy and continued cervical cancer screening.

Measurable outcomes include completion of routine cancer screenings, improved arm mobility through increased physical activity, and dietary changes (e.g., increased intake of fruits, vegetables, and healthy fats). If used as a checklist, the model could potentially support consistent anticipatory guidance and documentation. Less measurable but important outcomes include clinician confidence in survivorship care and patient trust that her breast cancer history informs ongoing care.

These topics supported by care coordination and the main ideas will need to be tested within clinical scenarios in oncology and primary care settings to ensure they capture desired information. Future intervention testing and implementation research could potentially be guided by the model after its thorough vetting. What we know now, however, is that if a CPG or corresponding framework about care for PHBC is not user-friendly, non-oncology specialists (i.e., primary care) will most likely not use it. Further, non-oncology specialists may resort to their previous experiences treating PHBC, or worst case not consider factors relevant to history of breast cancer. We have identified topics and main ideas that are supported by care coordination and could serve as the basis or checklist for anticipatory guidance. As such, we potentially provide a tangible starting point for addressing PHBC needs and concerns in primary care.

To the best of our knowledge, breast cancer-specific survivorship care CPGs, such as those published by Runowicz et al. on behalf of the American Cancer Society and the American Society of Clinical Oncology, have not been updated since their first publication in 2016. Equally problematic is that the supporting evidence may rely too heavily on non-randomized control trial (RCT) studies [24, 25]. Pan et al. (2018) report that few PHBC studies include a control group of people who are cancer-free, hindering the ability to distinguish between effects of aging and cancer treatment’s contribution to the aging process. Reliance on lower levels of evidence and few RCTs to continue to revise CPGs does not help healthcare providers in primary care remain aware of current recommendations for symptom management and surveillance activities, nor help them to effectively engage with PHBC to identify how changes brought on by aging impact the topic areas we identified.

Finally, risk management of breast cancer recurrence is complex and guidance about surveillance for new cancers in this population can be inconsistent [68]. That said, primary care clinicians are going to identify new and recurring cancers, but perhaps not during routine care or physical exams. Analysis by Beltran-Bless et al. (2023) found that most local recurrences of breast cancer in their study were identified during non-routine outpatient visits where patients reported signs and symptoms. Likewise, routine mammography played a key role in identifying ipsilateral recurrences [70]. Only two of the 206 recurrences evaluated in this study were identified by healthcare providers during regularly scheduled follow-up visits. Saltbæk et al. also described patterns of recurrence in a Danish follow-up program for PHBC [71]. While they found a higher percentage of recurrences attributable to general practitioners or other specialists (47%), they similarly found that the majority of recurrences were discovered via patient-reported symptoms (88%). Findings such as these lend support to the three topics supported by care coordination we identified in our synthesis. Each topic includes symptom assessment and discussions framed with a breast cancer survivorship lens meant to inform and alert primary care to the need for additional work up or referrals.

## Limitations

Our systematic review of CPGs that are clinically relevant to PHBC comes with strengths and limitations. We were diligent with our protocol and assessment of CPGs to be included. We did not anticipate inclusion of 21 different records, and it is worth noting that the number of initial results was somewhat staggering as was the method of guideline creation and reporting. Readers and consumers of guidelines must proceed with caution when interpreting publications as “clinical practice guidelines.” CPG clarity varied because of the diverse nature of how the guidelines were presented or published. In several cases, published CPG articles addressed a portion of a larger, algorithm-based CPG which may have included more detailed information. Also, few CPGs were stand-alone documents. Most contained links to supplements and related CPGs from the same organization and referred to online standard operating procedures for the organization’s CPG methodology and updates. Thus, we found few CPGs that could function as stand-alone documents amenable to immediate data extraction or quality appraisal, i.e., we had to take extra steps to obtain a comprehensive understanding of the CPG.

The lower-rated AGREE II domain related to Stakeholder Involvement might suggest oncology-centric recommendations, meaning the topics and main ideas may not sufficiently address PHBC issues and concerns, thereby limiting the generalizability of the model in its current state. The lower scores in the AGREE II domain of Editorial Independence could introduce additional bias into the model. For these reasons, the topics and main ideas presented in Fig. 4 should be evaluated with careful consideration of the real-world clinical context and patient populations in which they may be used. The additional evaluation of the model would adjust its current form and likely mitigate any bias or inconsistencies that could potentially be present because of these varied AGREE II domain scores.

Conducting a comprehensive and up-to-date systematic review of CPGs was challenging due to the rate of CPG updates. Three guidelines had to be re-appraised and re-extracted because they were updated before the review was complete [54, 56, 64]. Although systematic reviews typically have a cut-off date for article retrieval, these CPG updates contained more pertinent guidelines for cancer survivorship compared to the previous versions, and for this reason were included in the review. Future systematic reviews of CPGs must balance the need for the most current information against the practical realities and available resources for conducting a systematic review.

Our synthesis process was conducted by similarly disciplined, US-based, PhD-prepared nurse scientists, one being an advanced practice nurse, another a former oncology nurse, and another a PHBC. Certainly and importantly, before exploring the three topic areas and care coordination further, additional examination of the topics and main ideas would need to be completed by experts and stakeholders outside the research team. The included CPGs are geographically diverse, although the majority do originate from the USA. As such, the transferability of our model to non-US settings would depend greatly on the country-specific, current state of breast cancer survivorship care delivery, yet could still potentially serve as a starting point for other settings trying to bridge gaps in breast cancer survivorship care. Even in the USA, the applicability of our findings would be influenced by local healthcare structures, insurance coverage, any current cancer survivorship models already in place, and whether those models were oncology or primary care driven.

## Clinical implications

Our work here has potential implications for PHBC and their clinicians. As such, our challenges are not unique. The big questions are as follows: if the three topic areas we have identified supported by care coordination test well in additional research and controlled clinical scenarios (i.e., research settings with PHBC and primary care clinicians), how will that success be communicated beyond the research setting? What is the best way to implement CPGs or care models specific to PHBC? This remains problematic when many CPGs are written for a broad audience yet published in oncology-specific journals or settings. Similarly, frequent updates to type-specific cancer survivorship guidelines can get lost in the vast amount of data that must be managed by primary care clinicians. Care for PHBC is still fragmented, even as years of survivorship increase. Importantly, as the number of PHBC increases, their health care will likely be provided by primary care clinicians. Tailoring breast cancer survivorship care guidelines and models to primary care use seems imperative. Our long-term objectives are to accept the challenges and facilitate this process.

## Conclusion

Our systematic review identified main topics that could be considered in streamlining the CPG guidance for PHBC survivorship care: risk for recurrence, monitoring for new cancer, and fertility, reproductive, endocrine, and sexual health. The three topics supported by care coordination could form a usable model for PHBC survivorship care. These topics would be considered alongside care coordination, but all would need to be tested and vetted before overstating any conclusions. We advocate for future research that evaluates our proposal as part of efforts to refine best practices in providing appropriate long-term cancer survivorship care to PHBC. Notably, we highlight the importance of simplifying and standardizing breast cancer survivorship care so that it could be readily implemented in the primary care setting.

## Data Availability

Data that support the findings of this systematic review and synthesis (e.g., additional tables) may be made available by contacting the first author.
